# Supplementary material for: Adolescent choices and caregiver roles: Understanding individual and interpersonal influences on sexual decision-making in South Africa
Source: PLOS Glob Public Health. 2024 Dec 9;4(12):e0003569. doi: 10.1371/journal.pgph.0003569 (PMC11627436; doi:10.1371/journal.pgph.0003569)
Supplement: S1 Checklist — (PDF) [file pgph.0003569.s003.pdf]

Consolidated criteria for reporting qualitative studies (COREQ): 32-item checklist for manuscript in title:

"Adolescent choices and caregiver roles: Understanding individual and interpersonal influences on sexual decision-making in South Africa"

No	Item	Guide questions/description	Response
**Domain 1: Research team and reflexivity**			
Personal Characteristics			
1.	Interviewer/facilitator	Which author/s conducted the interview or focus group?	Done<br>It is mentioned in the method lines 169 to 176
2.	Credentials	What were the researcher's credentials? *E.g. PhD, MD*	Done<br>It is mentioned in the method lines 169 to 176
3.	Occupation	What was their occupation at the time of the study?	Done<br>It is mentioned in the method lines 169 to 176
4.	Gender	Was the researcher male or female?	Done<br>It is mentioned in the method lines 169 to 176
5.	Experience and training	What experience or training did the researcher have?	Done<br>It is mentioned in the method line 171
Relationship with participants			
6.	Relationship established	Was a relationship established prior to study commencement?	No
7.	Participant knowledge of the interviewer	What did the participants know about the researcher? e.g. *personal goals, reasons for doing the research*	Participants and interviewers maintained professional relationships up until the end of the study, mentioned in methods, lines174-176
8.	Interviewer characteristics	What characteristics were reported about the interviewer/facilitator? e.g. *Bias, assumptions, reasons and interests in the research topic*	None
**Domain 2: study design**			
Theoretical framework			
9.	Methodological orientation and Theory	What methodological orientation was stated to underpin the study? *e.g. grounded theory, discourse analysis, ethnography, phenomenology, content analysis*	*thematic analysis*<br>It is mentioned in the method section, lines 189 to 194
Participant selection			
10.	Sampling	How were participants selected? *e.g. purposive, convenience, consecutive, snowball*	*Convenience*<br>It is mentioned in the method section, lines 151 to 153
11.	Method of approach	How were participants approached? e.g. *face-to-face, telephone, mail, email*	*In-person*<br>It is mentioned in the method section, lines 156
12.	Sample size	How many participants were in the study?	17 adolescents and 19 caregivers<br>It is mentioned in the results section, lines 215 & 223
13.	Non-participation	How many people refused to participate or dropped out? Reasons?	0

			Not mentioned, assumed by sampling technique used.
Setting			
14.	Setting of data collection	Where was the data collected? e.*g. home, clinic, workplace*	*Research centre*<br>It is mentioned in the method section, line 137
15.	Presence of non-participants	Was anyone else present besides the participants and researchers?	0<br>Not mentioned, assumed by sampling technique used.
16.	Description of sample	What are the important characteristics of the sample? *e.g. demographic data, date*	Done<br>It is mentioned in the method lines 136 to 153
Data collection			
17.	Interview guide	Were questions, prompts, guides provided by the authors? Was it pilot tested?	Done<br>It is mentioned in the method lines 163 to 169
18.	Repeat interviews	Were repeat interviews carried out? If yes, how many?	No<br>Not mentioned
19.	Audio/visual recording	Did the research use audio or visual recording to collect the data?	Yes<br>It is mentioned in the method section, lines 184 to 187.
20.	Field notes	Were field notes made during and/or after the interview or focus group?	Yes<br>It is mentioned in the method section, lines 184 to 187.
21.	Duration	What was the duration of the interviews or focus group?	It is mentioned in the method section, lines 160 to 161.
22.	Data saturation	Was data saturation discussed?	No<br>Limitations mentioned, lines 425 to 430.
23.	Transcripts returned	Were transcripts returned to participants for comment and/or correction?	No<br>Not mentioned
**Domain 3: analysis and findings**			
Data analysis			
24.	Number of data coders	How many data coders coded the data?	There was one coder and two reliability checkers.<br>Mentioned in methods section, lines 189 to 192.
25.	Description of the coding tree	Did authors provide a description of the coding tree?	Mentioned lines 189 to 192, which informed theme levels provided in Table 1.
26.	Derivation of themes	Were themes identified in advance or derived from the data?	Mentioned lines 189 to 192, which informed theme levels provided in Table 1.
27.	Software	What software, if applicable, was used to manage the data?	It is mentioned in the method section line 189
28.	Participant checking	Did participants provide feedback on the findings?	No<br>Not mentioned
**Reporting**			
29.	Quotations presented	Were participant quotations presented to illustrate the themes / findings? Was each quotation identified? e.*g. participant number*	Done
30.	Data and findings consistent	Was there consistency between the data presented and the findings?	Done
31.	Clarity of major themes	Were major themes clearly presented in the findings?	Done

32.	Clarity of minor themes	Is there a description of diverse cases or discussion of minor themes?	Table 1 shows the socioecological model (SEM) level, and the themes that emerged for the two SEM levels
