# Supplementary material for: Adolescent choices and caregiver roles: Understanding individual and interpersonal influences on sexual decision-making in South Africa
Source: PLOS Glob Public Health. 2024 Dec 9;4(12):e0003569. doi: 10.1371/journal.pgph.0003569 (PMC11627436; doi:10.1371/journal.pgph.0003569)
Supplement: Attachment — Submitted filename: Response to Reviewers.pdf [file pgph.0003569.s006.pdf]

THE AURUM INSTITUTE
SOUTH AFRICA
Registration No. 2021/785844/08

Aurum House
The Ridge, 29 Queens Road
Parktown 2193
South Africa

PostNet Suite300
Private Bag X30500
Houghton 2041
South Africa

Tel: +27 (0) 10 590 1300
Fax: +27 (0) 866 180 268
Website: www.auruminstitute.org

08 September 2024

Dr Marie A. Brault
Academic Editor
PLOS Global Public Health

REFERENCE: PGPH-D-24-01646

RESPONSE LETTER

Dear Dr Brault,

We thank the editors and reviewers for their thoughtful comments and speedy response of our manuscript entitled "Adolescent choices and caregiver roles: Understanding individual and interpersonal influences on sexual decision-making in South Africa". Below is a point-by-point response where changes have been made.

Editor's Comments:

1. The reviewers and I found the manuscript to be generally well-written, and feel it provides interesting insights into the dynamics of caregiver/youth conversations and education concerning sexual health.

   Response: Thank you, this is encouraging.

2. However, there are a few areas that could use some additional clarification and expansion. Additional information on the qualitative sample (how recruitment occurred, whether the caregivers are linked to the youth participants or not, etc.). In addition, please see and address Reviewer 3's comments concerning the language and areas that should be elaborated on, if possible.

   Response: These are noted, and we provide below responses to each of the clarifications raised.

First Reviewers Comments:

Abstract section:

1. I would advise that you structure the abstract in to four sections i.e. Introduction, Methods, Results and Conclusions.

Response: We applied the instructions provided on web page https://journals.plos.org/globalpublichealth/s/submission-guidelines, where it indicates "While the Abstract is conceptually divided into three sections (Background, Methodology/Principal Findings, and Conclusions/Significance), do not apply these distinct headings to the Abstract within the article file."

2. Sentence on line 42 and 43 is not clear maybe it could be,

   Thematic analysis revealed that while adolescents had access to sexual education from various sources, this knowledge did not translate into healthy sexual decision-making.

   Response: Thank you for this suggestion. We have edited the text to include this revised sentence.

3. The term sexual activity is not clearly understood in the entire manuscript. Does sexual activity mean sexual matters (issues), or intercourse please be clear.

   Response: To clarify this information, we changed sexual activity to behaviour throughout the manuscript. For reference, see lines 45, 46, 276, and 356 in clean manuscript.

4. You should have like a maximum of 5 key words just after the conclusion on the abstract.

   Response: We have reduced the number of key words to include, "Caregiver roles, Adolescent choices, sexual behaviour, HIV, South Africa".

5. You should move authors' contributions after the abbreviations' section.

   Response: See https://journals.plos.org/globalpublichealth/s/file?id=3fac/PLOS%20Affiliations%20Formatting%20Guidelines.pdf, the indication in text block seem to indicate this section should be included in the title page. This though can be rectified in the final proof version ahead of print.

Introduction section:

1. On line 86. The HIV burden among sub-Saharan African (SSA) adolescents . . . make sub upper case.

   Response: Corrected to "*The HIV burden among Sub-Saharan African (SSA) adolescents is a significant public health concern with adolescent girls and young women (AGYW) aged 15-24 years accounting for 63% of new HIV infections in the region*".

2. On line 108. The role of discussing sensitive matters . . . what do you mean by sensitive matters? These matters could be family, political, religious, economic, etc. please be clear.

   Response: We have revised this information to read as follows "*The role of discussing sexual matters with adolescents generally becomes the responsibility of the female caregivers, as*

male caregivers are generally perceived as being absent, difficult, uncomfortable, and incapable of fostering close bonds with children". We have further made changes to other sections in the manuscript where the word "sensitive" is used.

3. On line 127. . . a qualitative exploratory study to understand the factors that influences sexual .. . The sentence should read . . . a qualitative exploratory study to understand the factors that influence sexual…

   Response: Corrected to "*Therefore, we conducted a qualitative exploratory study to understand the factors that influence sexual decision-making from the perspectives of adolescents and their caregivers in Rustenburg, South Africa*".

4. Please include a geographical scope in your concluding statement of the introduction. E.g. Therefore, we conducted a qualitative exploratory study to understand the factors that influences sexual decision making from the perspectives of adolescents and their caregivers in . . .

   Response: I included Rustenburg, South Africa and the revised sentence reads as follows: "*Therefore, we conducted a qualitative exploratory study to understand the factors that influence sexual decision-making from the perspectives of adolescents and their caregivers in Rustenburg, South Africa*".

Method section:

1. Study design and setting should be study design and settings.

   Response: Corrected, see line 129

2. On lines 137 and 138 you state that, Adolescents between the ages of 12-19 years constitute 14.6% of the population and have a high prevalence of HIV. Please mention this high prevalence.

   Response: This was included in error and remained from a prior version, our apologies. We have removed this part of the sentence and corrected the sentence to "*Rustenburg is one of the fastest growing cities in the Northwest Province where platinum mining is the dominant industry, with an approximate population of 562,031 in 2022, where 25% being 15 years old or younger*".

3. Paragraphs two and three in the methods section are disjointed. They do not speak to the introduction nor to the study title. Please rewrite this section basing on the current objectives not anything else.

   Response: We have included the details to the parent study to provide context on how participants were invited to participate in the focus group discussions. Since direct recruitment was not done for this analysis, we provided detail in how we reached participants for the qualitative study. In the first paragraph under the study design and settings section we indicate

"*This qualitative project was embedded in a parent observational prospective study that took place at the Aurum Rustenburg Research Centre, South Africa*".

4. How many FGDs did you conduct with Adolescents and with Caregivers also describe their composition. From the text one guesses that they were four 2 with adolescents and 2 with caregivers. FGDs being only 2 for each of the categories seem to have compromised validity and reliability of your findings. This being a qualitative study where data collection is collected up to saturation point mere two FGDs with each of the categories makes the findings less dependable.

   Response: In total we conducted four FGDs, two with adolescent participants and two with caregivers. The FGDs included a diverse group of individuals that included different ages, genders and relationships to the caregivers. A measure that we put in place to monitor saturation was to review probes in real time and adjust probing to ensure that we gathered adequate information across all participants. Although we conducted 2 FGDs per category of participants, we feel that the participant attributes, variation in experiences and our method of monitoring saturation supported the reliability of our results. We include further text to support the description of data collection in lines 165-166, "*To measure saturation, we reviewed probes in real time and adjust probing to ensure that we gathered adequate information across all participants*";
   and to expand on the limitations in lines 460-462 "*Due to the design and provisions of the parent study, the data collected for this study was limited to four FGDs, and therefore we cannot confirm nor conclude saturation of emergent themes*".

5. In lines 159 – 161, you state that FGD guides (attached S1 and S2) were used to explore the adolescent and caregiver knowledge of sexual and reproductive health, and factors contributing to sexual and reproductive health behaviour. Yet the objective of this study was to understand the factors that influences sexual decision making from the perspectives of adolescents and their caregivers (refer to lines 126 - 128). This means that you collected somewhat different data from your objective. Please rework on this section very well. Be consistent and stick to the objective of this Manuscript.

   Response: This is a valid point. The guides present several topics, thus we have corrected to include the main topics in the guide related to this work. We have added further explanation in lines 160-163: "*The guides included many topics, including to understand the factors that influence sexual decision-making from the perspectives of adolescents and their caregivers and recommendations which could encourage adolescents to speak honestly about their sexual behaviours*".

6. Please describe the inclusion and exclusion criteria of the adolescents and the care givers into the study.

   Response: For clarity we have added further detail to distinguish eligibility for the parent study and the criteria used for FGDs. This reads as follows: "*Adolescents who met the eligibility criteria for the parent study and the caregivers (parent or guardian) of 15- to 17-year-old eligible participants were invited to take part in focus group discussions (FGD).*

FGDs were conducted within three months after enrolling into the main study, between April to July 2018. Adolescents and caregivers were selected using convenience sampling on a first come basis, considering their availability for a scheduled date within the 3-month window, and concluded once a quota of 8 to 10 participants were reached".

7. In line 194 – 195 you state that, all participants were reimbursed ZAR150.00 (about $8.20 USD) for participation in the FGDs. This imply means you paid them for taking part in the study. I suggest you revise this statement to mean that all participants were given a small token of appreciation ZAR150.00 (about $8.20 USD) for sparing their time to take part in the study.

   Response: Correction made to indicate "*All participants were given a small token of appreciation ZAR150.00 (about $8.20 USD) for sparing their time to take part in the study and to compensate for any expenses they may have had*".

Results section:

1. In line 203 you state 17 adolescents participants in the study but in lines 152 – 154 you state that, two in-person FGDs were conducted with adolescents and two with caregivers following enrolment into the main study and before a month-3 follow-up visit. Each FGD comprised 8-10 participants. This is a glaring contradiction and poses a number of questions on researcher team's honesty.

   Response: We planned to have 8-10 participants per FGD, aiming for a total of 20 participants. We were able to include 17 participants in total across the 2 FGDs staying within the proposed sample size. The revised sentence reads as follows: "*In total , 17 adolescents (13 females and 4 males) participated in the two FGDs, with median age 17-years (Interquartile range (IQR) 16-18 years)*".

2. Is the data of 2018 still applicable and publishable in the last half of 2024 in order to inform policy or any other development agenda? Haven't the findings of this study been overtaken by events considering the time differences?

   Response: We believe the need to understand adolescent sexual decision-making remains given the continued HIV burden faced. We have included further comment to this aspect in lines 495-498: "*While the data is collected in 2018, the continued HIV burden among AGYW, represents the need to understand factors that may continue to influence adolescent sexual decision-making and to inform prevention strategies that can be used to reduce adolescents' exposure for HIV*".

Discussion section:

1. The discussion sections flow very well. However, I recommend that the research teams adopt the art of using short sentences as compared to long ones. This makes the reader follow the story clearly without having to keep rereading the sentences.

Response: Noted. We have made changes to some sentences to make shorter and enhance reading flow.

2. Please proofread the entire manuscript to avoid obvious errors and punctuation issues.

   Response: We have reviewed the entire manuscript and made further changes where needed.

3. I also suggest that a section of the study limitations be included in the Manuscript.

   Response: Lines 459-471 comprise the study limitations we have included.

4. A section on data availability should also be included in the Manuscript.

   Response: We have now included link to accessing the data within the supporting information section, see line 653 (https://doi.org/10.25382/iavi.26540635.v1).

Conclusion section:

1. This is well articulated and emanates from the study findings.

   Response: Thank you. Trusting this add evidence to driving policy and inform future intervention design.

References section:

1. These should be the last section of the manuscript there after any appendices can follow.

   Response: We follow the layout as indicated in URL https://journals.plos.org/globalpublichealth/s/submission-guidelines, where references are indicated to follow acknowledgement section.

Second Reviewers Comments:

1. In the abstract section, clarify the statement: "Thematic analysis revealed that while adolescents had access to sexual education from various sources, where the knowledge does not translate into healthy sexual decision-making.".

   Response: We have edited this sentence to "*Thematic analysis revealed that while adolescents had access to sexual education from various sources, this knowledge did not translate into healthy sexual decision-making*".

2. In the study design and setting section, provide more details on the specific methods used for convenience sampling to enhance transparency and replicability.

   Response: We have added further description to the convenience sampling: "*Adolescents and caregivers were selected using convenience sampling on a first come basis, considering their availability for a scheduled date within the 3-month window, and concluded once a quota of 8 to 10 participants were reached*".

3. While the discussion section offers valuable insights, it needs improvements for better clarity and depth. Integrate the findings more effectively with existing literature to contextualize the results, such as referencing previous studies that identify similar barriers like poor communication between adolescents and caregivers.

   Response: We have included text in the relevant paragraph, and reads as follows: "*Caregivers knew the benefits of being more supportive but felt helpless as they lacked the knowledge and ability to engage in sexual behaviour discussions with their adolescents. Similar findings have been reported in SSA, where the ability of caregivers to engage with adolescents in sexual health topics are strained due to caregivers' inabilities to discuss sex, generation gaps, proscriptive socio-cultural beliefs and moralistic and religious views (12–14,25). Targeted interventions engaging with caregivers to strengthen skills and comfort in having sex topic discussions and providing support for their children in their sexual health choices is a pressing need in SSA and South Africa (12–14,25). A South African study identified that increased positive caregiving and caregiving supervision were protective factors to adolescent engaging in HIV risk behaviours (26). Further, caregiver support could enhance adolescent self-efficacy and improve self-esteem toward healthy sexual decision-making (19)*".

4. The discussion on the intention-behaviour gap and gain-framed messages requires more detailed explanations of practical implementation strategies. Additionally, the section on male caregivers should include specific interventions or programs that have successfully redefined traditional roles and how these could be adapted in the study context.

   Response: We understand this concern raised. Since we had not investigated the intention-behaviour gap in our study and it remains unknown in SSA we have resolved to remove this aspect in the discussion section. Regarding aspects of gain-framed messaging and its possible use in promoting healthy adolescent decision-making, we have leveraged on literature available, as this aspect too remains underrepresented for Sub-Saharan Africa. We have resolved to include the following paragraph, cognisant that we had not investigated this strategy in our study. The paragraph reads as follow: "*Another strategy to improve translating knowledge toward healthy sexual decision-making, is in using contextually relevant gain-framed messaging to emphasize the benefits of engaging in behaviours which have positive health outcomes (33). Although adolescents receive information and have knowledge of sexual behaviours, they struggle to make immediate healthy choices for their sexual health. The standard educational material provided to adolescents possibly does not emphasise the benefits for individual well-being based on immediate choices around their sexual behaviour. Gain-framed messaging (benefits of healthy sexual decision choices) instead of loss-framed messaging (harms of not making healthy sexual decision choices) can have motivating results in healthy sexual decision choices (33,34). In addition, reaching adolescents and caregivers through television and social media has broader potential in propagating gain-framed messaging (35). The role of gain-framed messaging in promoting healthy sexual decision-making is not known in SSA (35) and represents opportunity to investigate this strategy in future*".

   Regarding the role male caregivers can have in supporting their adolescent children we have added text. This reads as follows "*Interventions with targeted approaches in improving male*

caregiver skills to nurture relationships with children, improving nuclear family relationships, empowering males with knowledge and skill to refrain from violence at home, and engaging men as partners in their female partners sexual reproductive and maternal health have produced encouraging results. Increasing male caregiver shared responsibility in domestic chores and caregiving of children have improved caregiver gender-equality inadequacies, enhances nuclear family relationships and have reduced male dominance in decision-making (28)".

5. The limitations section should be more thorough, discussing the potential impacts of these limitations on the findings and suggesting ways to address them in future research. Propose strategies for including a more diverse participant pool or involving external stakeholders to provide a more comprehensive understanding.

   Response: We have revised the limitations section to read as follows: "*Our study was limited to participants who were already accessing services in a research setting, and an unequal representation of both male adolescents and caregivers. Due to the design and provisions of the parent study, the data collected for this study was limited to four FGDs, and therefore we cannot confirm nor conclude saturation of emergent themes. We also did not include the perspectives of external stakeholders such as community leaders, policy makers, and potential influential role models within the community. Thus, this study is restricted in its ability to capture the perspectives of the broader community, including capturing the perspectives of more adolescents and caregivers, and the findings should be considered within these limitations. We are not able to determine the degree of community proscription in having sexual behaviour discussions between adolescents and caregivers, and if this is contextually related to specific community groups. Future studies could include the voices of more adolescent and caregiver participants, and the inclusion of other members of communities and societies*".

6. The conclusion effectively summarizes the key findings of the study but could be enhanced for clarity and impact. Here are some suggestions:
   - Emphasize practical implications by highlighting how the findings can be directly applied to develop specific interventions or programs.
   - Include future research directions to suggest areas for future research to build on the study's findings.

   Response: We have made several changes following these helpful suggestions, and that of reviewer 3. We have made these cognisant of the study findings and to include aspects emerging from the study only. The section now reads as follows: "*Overall, the study provides context of the multiple individual and interpersonal factors influencing adolescent sexual decision-making and behaviour, and the need to provide a supportive environment which enables sexual behaviour choices for healthy sexual and reproductive outcomes. Such supportive environments include protective measures which seeks to prevent marginalizing sexual behaviour choices at home and in community, and enables adolescents the agency to make the choices with knowledge of behaviours which are likely to have positive health sexual and reproductive outcomes (36). The study provides valuable insights to the critical role caregiver-adolescent communication has in promoting healthy sexual decision-making. The*

findings underscore the importance of implementing targeted, culturally relevant interventions which include sexual education and skill package to equip both male and female caregivers to engage in conversations on sexual behaviour. The study as well highlights the potential in including gain-framed messaging within intervention packages which could enable adolescents' agency to translate knowledge towards healthy sexual choices. Lastly, the study adds to existing evidence highlighting the potential benefits in engaging men in improving their nurturing and gender-equitable roles at home, and the powerful response they can have in improving health and social outcomes for their family".

Third Reviewers Comments:

1. Review of manuscript: Adolescent choices and caregiver roles: Understanding individual and interpersonal influences on sexual decision-making in South Africa
   This is a well written paper overall and addresses an important topic. I have a few specific comments and some more general ones for the authors' consideration. I have also included some suggestions for additional literature which I think could help to strengthen some of the areas of the discussion.

   Response: Thank you for these. We have considered these in our responses below and in general to further shaping the manuscript.

2. Line 35: "South African adolescents are at-risk for HIV infection" – through sexual transmission. Consider adding this clarification, otherwise the next sentence feels slightly non secateur.

   Response: We have corrected to "*South African adolescents are at-risk for HIV infection due to engaging in high-risk sexual behaviours*".

3. Line 37: The adolescents' caregivers? Were they paired?

   Response: Due to the word count for abstracts we had not included this level of information. We provide further clarity within the methods section: "*Caregivers were not necessarily the caregivers of adolescent participants who were included in FGDs*".

4. Line 42: something missing in this sentence? – is incomplete: "where the knowledge does not translate into healthy sexual decision-making….??".

   Response: We have edited this sentence to "*Thematic analysis revealed that while adolescents had access to sexual education from various sources, this knowledge did not translate into healthy sexual decision-making*".

5. Line 364: what is a "good choice" ? perhaps a risk of being morally laden by suggesting that choices are good or bad. Perhaps rephrase to focus on the outcomes of the choice resulting in positive or negative health outcomes.

Response: Valid point. We have made correction to "*Making choices to improve one's sexual health outcomes is the foundation for positive sexual decision-making*".

6. Line 366: "knowledge of sexual behaviour" ? or knowledge of risks associated with sexual behaviours and risk reduction / avoidance / prevention strategies?

   Response: We have corrected to read as follows: "*Through our qualitative investigation conducted in South Africa, we found that adolescents had adequate knowledge of risks associated with certain sexual behaviours but do not translate this knowledge into choices for healthy sexual outcomes*".

7. Line 369: enabled informed decision making?

   Response: Correction reads as follows: "*This barrier limited the type of support that caregivers could provide to their adolescents to enable informed decision-making*".

8. Lines 370-371: more than just their ability to communicate effectively about SRH - evidence suggests that lack of knowledge and skills, combined with generational and educational gaps between parents and adolescents, also contribute to parents' sense of disempowerment, lack of self- efficacy, and reluctance to discuss SRH.

   Response: Thank you for the suggested text. Correction reads as follows: "*While caregivers knew of the benefits of communicating and supporting their adolescents, evidence suggests that lack of knowledge and skills, combined with generational and educational gaps between caregivers and adolescents, also contributed to caregivers' sense of disempowerment, lack of self-efficacy, and reluctance to engage in communication in healthy sexual choices*".

9. Line 388: what is meant by "caregiver intervention" ?

   Response: We have added further text to clarify. Correction reads as follows: "*Similar findings have been reported in SSA, where the ability of caregivers to engage with adolescents in sexual health topics are strained due to caregivers' inabilities to discuss sex, generation gaps, proscriptive socio-cultural beliefs and moralistic and religious views (12–14,25)*".

10. General comments:
    10.1 In the findings section, the authors outline factors at the individual and interpersonal / relationship levels of the SEM framework. It would be interesting to know whether factors at the socio-cultural / contextual level came up – as social and cultural guidelines and proscriptions around sexuality communication between adolescents and caregivers are very deeply rooted, particularly in the sub-Saharan African setting.

    Response: This aspect was evident in caregivers not being able to translate their knowledge and experiences to their children due to the taboo nature in having sexual topic discussions with children, and likewise the adolescents felt the same (see lines 342-343, "*However, some caregivers and adolescents avoided discussions on sexual behaviour due to the historically taboo nature of talking about sex or other sexuality topics with minors*").

The study data is though limited and with the inclusion of more adolescent and caregiver participants this could have been explored further. We as well had not included other member of the community to understand the degree of this belief and if it associated with all in the community, or specific to certain groups in the community. We have rephrased the limitations section to read as follows: "*Our study was limited to participants who were already accessing services in a research setting, and an unequal representation of both male adolescents and caregivers. Due to the design and provisions of the parent study, the data collected for this study was limited to four FGDs, and therefore we cannot confirm nor conclude saturation of emergent themes. We also did not include the perspectives of external stakeholders such as community leaders, policy makers, and potential influential role models within the community. Thus, this study is restricted in its ability to capture the perspectives of the broader community, including capturing the perspectives of more adolescents and caregivers, and the findings should be considered within these limitations. We are not able to determine the degree of community proscription in having sexual behaviour discussions between adolescents and caregivers, and if this is contextually related to specific community groups. Future studies could include the voices of more adolescent and caregiver participants, and the inclusion of other members of communities and societies*".

10.2 Peer pressure – the authors describe the influence of social media etc – but it would seem that some of this relates to peer pressure, desire to conform, and perceptions of what other adolescents are doing. Related to this - what about adolescents' desire to be fashionable etc. Evidence suggests that materialism and pressure to conform and achieve social status influences adolescent risk behaviours.

Response: This is a valid point, and one aspect we had identified and omitted to include in our findings. Addition reads as follows: "*Peer pressure and desires to conform to activities their peers engage in were other influences in adolescent choices to engage in sexual behaviours. The drive for materialistic rewards, particularly if they are poor, influences adolescent engagement in transactional sexual relationships (29,30). Friends, peers and sexual partners can be influential in adolescents engaging in sexual behaviours, sexual abuse, alcohol and illicit drug use. While some participants reflected on positive influences friends, peers and sexual partners can have, dominant concern to the negative influences was shared by both adolescents and caregivers*".

10.3 One aspect that could be expanded on pertains to the ways in which power inequities and agency disparities impact the ability of adolescent girls to make decisions about their SRH and top adopt behaviours that are protective for their sexual health.

Response: Thank you for the suggested literature and flagging our omission of this aspect in discussion section. Addition reads as follows: "*These findings provide insights to the agency adolescents have in choosing to engage in sexual behaviours. Across SSA, adolescents' risks for becoming sexually active is not by choice alone and have multiple intrinsic and extrinsic influences (31,32). While adolescents conform less to traditional and cultural gender roles, relationship power inequities and hegemonic masculine beliefs continue to fuel beliefs in male dominance (32). All of which have poor outcomes for adolescents, specifically AGYW, in defining their agency toward positive choices for their sexual and reproductive health (32).*

The evidence provided here and in literature resonates the need for enhanced community and social support systems that transcend across SEM levels for enduring behaviour change (19)".

10.4 Relating to my comment about what constitutes a "good choice" – in my opinion, the authors could pay closer attention to the language and terminology used around risk – to ensure that the language avoids any moralising. There is literature about the 'discourse of risk' (see the Shoveller reference below). I would urge the authors to consider the framing of adolescent choices, risk engagement and decision making to ensure that the focus is on health outcomes.

Response: We have reviewed the manuscript to revise sections which may have moralising meaning. We have as well included text further in discussion as can be seen in responses 10.2 and 10.3 and in the conclusion section: "*Overall, the study provides context of the multiple individual and interpersonal factors influencing adolescent sexual decision-making and behaviour, and the need to provide a supportive environment which enables sexual behaviour choices for healthy sexual and reproductive outcomes. Such supportive environments include protective measures which seeks to prevent marginalizing sexual behaviour choices at home and in community, and enables adolescents the agency to make the choices with knowledge of behaviours which are likely to have positive health sexual and reproductive outcomes (36)*".

Please do not hesitate to contact me if you require any additional information.

Kind regards,

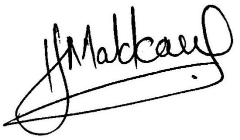

Digitally signed by Heeran Makkan
Date: 2024.09.08 20:04:55 +02'00'

Heeran Makkan
Senior Programme Manager- HIV Treatment and Prevention
Clinical Research Division, The Aurum Institute
Email: hmakkan@auruminstitute.org
Mobile: +27 82 302 2044
