# Supplementary material for: Adolescent choices and caregiver roles: Understanding individual and interpersonal influences on sexual decision-making in South Africa
Source: PLOS Glob Public Health. 2024 Dec 9;4(12):e0003569. doi: 10.1371/journal.pgph.0003569 (PMC11627436; doi:10.1371/journal.pgph.0003569)
Supplement: Attachment — Submitted filename: Manuscript PGPH-D-24-01646.docx [file pgph.0003569.s004.docx]

**Manuscript #:** PGPH-D-24-01646

**Title:** Adolescent choices and caregiver roles: Understanding individual and interpersonal influences on sexual decision-making in South Africa
**Authors:** Heeran Makkan; Yvonne Wangui Machira; Funeka Mthembu; Omphile Masibi; Thuso Molefe; Pholo W. Maenetje; Vincent Muturi-Kioi; Matt A. Price; Vinodh A. Edward; Candice Chetty-Makkan

**Article type:** Research Article

**Abstract**

1. I would advise that you structure the abstract in to four sections i.e. Introduction, Methods, Results and Conclusions
2. Sentence on line 42 and 43 is not clear may be it could be,
3. Thematic analysis revealed that while adolescents had access to sexual education from various sources, this knowledge did not translate into healthy sexual decision-making.
4. The term sexual activity is not clearly understood in the entire manuscript. Does sexual activity mean sexual matters (issues) or intercourse please be clear
5. You should have like a maximum of 5 key words just after the conclusion on the abstract
6. You should move authors’ contributions after the abbreviations’ section

**Introduction**

1. On line 86. The HIV burden among sub-Saharan African (SSA) adolescents . . . make sub upper case
2. On line 108. The role of discussing sensitive matters . . . what do you mean by sensitive matters? These matters could be family, political, religious, economic, etc. please be clear
3. On line 127. . . a qualitative exploratory study to understand the factors that influences sexual .. . The sentence should read . . . a qualitative exploratory study to understand the factors that influence sexual .. .
4. Please include a geographical scope in your concluding statement of the introduction. E.g. Therefore, we conducted a qualitative exploratory study to understand the factors that influences sexual decision making from the perspectives of adolescents and their caregivers in . . . .

**Methods**

1. Study design and setting should be study design and settings
2. On lines 137 and 138 you state that, Adolescents between the ages of 12-19 years constitute 14.6% of the population and have a high prevalence of HIV. Please mention this high prevalence.
3. Paragraphs two and three in the methods section are disjointed. They do not speak to the introduction nor to the study title. Please rewrite this section basing on the current objectives not anything else.
4. How many FGDs did you conduct with Adolescents and with Caregivers also describe their composition. From the text one guesses that they were four 2 with adolescents and 2 with caregivers. FGDs being only 2 for each of the categories seem to have compromised validity and reliability of your findings. This being a qualitative study where data collection is collected up to saturation point mere two FGDs with each of the categories makes the findings less dependable.
5. In lines 159 – 161, you state that FGD guides (attached S1 and S2) were used to explore the adolescent and caregiver knowledge of sexual and reproductive health, and factors contributing to sexual and reproductive health behaviour. Yet the objective of this study was to understand the factors that influences sexual decision making from the perspectives of adolescents and their caregivers (refer to lines 126 - 128). This means that you collected somewhat different data from your objective. Please rework on this section very well. Be consistent and stick to the objective of this Manuscript.
6. Please describe the inclusion and exclusion criteria of the adolescents and the care givers in to the study
7. In line 194 – 195 you state that, All participants were reimbursed ZAR150.00 (about $8.20 USD) for participation in the FGDs. This imply means you paid them for taking part in the study. I suggest you revise this statement to mean that all participants were given a small token of appreciation ZAR150.00 (about $8.20 USD) for sparing their time to take part in the study.

**Results**

1. In line 203 you state 17 adolescents participants in the study but in lines 152 – 154 you state that, Two in-person FGDs were conducted with adolescents and two with caregivers following enrolment into the main study and before a month-3 follow-up visit. Each FGD comprised 8-10 participants. This is a glaring contradiction and poses a number of questions on researcher team’s honesty.
2. Is the data of 2018 still applicable and publishable in the last half of 2024 in order to inform policy or any other development agenda? Haven’t the findings of this study been over taken by events considering the time differences?

**Discussion**

1. The discussion sections flows very well. However I recommend that the research teams adopts the art of using short sentences as compared to long ones. This makes the reader follow the story clearly without having to keep rereading the sentences.
2. Please proof read the entire manuscript to avoid obvious errors and punctuation issues
3. I also suggest that a section of the study limitations be included in the Manuscript
4. A section on data availability should also be included in the Manuscript

**Conclusion**

1. This is well articulated and emanates from the study findings.

**References**

1. These should be the last section of the manuscript there after any appendices can follow
