# Supplementary material for: Adolescent choices and caregiver roles: Understanding individual and interpersonal influences on sexual decision-making in South Africa
Source: PLOS Glob Public Health. 2024 Dec 9;4(12):e0003569. doi: 10.1371/journal.pgph.0003569 (PMC11627436; doi:10.1371/journal.pgph.0003569)
Supplement: Attachment — Submitted filename: Comments.docx [file pgph.0003569.s005.docx]

This is an important, thorough, and well-written paper. I recommend the following minor revisions:

1. In the abstract section, clarify the statement: “Thematic analysis revealed that while adolescents had access to sexual education from various sources, where the knowledge does not translate into healthy sexual decision-making.”
2. In the study design and setting section, provide more details on the specific methods used for convenience sampling to enhance transparency and replicability.
3. While the discussion section offers valuable insights, it needs improvements for better clarity and depth. Integrate the findings more effectively with existing literature to contextualize the results, such as referencing previous studies that identify similar barriers like poor communication between adolescents and caregivers.
4. The discussion on the intention-behavior gap and gain-framed messages requires more detailed explanations of practical implementation strategies. Additionally, the section on male caregivers should include specific interventions or programs that have successfully redefined traditional roles and how these could be adapted in the study context.
5. The limitations section should be more thorough, discussing the potential impacts of these limitations on the findings and suggesting ways to address them in future research. Propose strategies for including a more diverse participant pool or involving external stakeholders to provide a more comprehensive understanding.
6. The conclusion effectively summarizes the key findings of the study but could be enhanced for clarity and impact. Here are some suggestions:
   - Emphasize practical implications by highlighting how the findings can be directly applied to develop specific interventions or programs.
   - Include future research directions to suggest areas for future research to build on the study's findings.
